# RNA splicing in bone diseases: mechanisms, pathogenesis and therapeutics

**DOI:** 10.3389/abp.2025.15819

**Published:** 2026-01-12

**Authors:** Linlin Zheng, Hui Sun, Ning Li, Lianqing Wang, Tianchu Li, Qiaoli Zhai

**Affiliations:** 1 Department of Plastic Surgery, Zibo Central Hospital, Zibo, China; 2 Department of Cardiac Intensive Care Unit, Zibo Central Hospital, Zibo, China; 3 Department of Ultrasound, Zibo Central Hospital, Zibo, China; 4 Translational Medicine Center, Zibo Central Hospital, Zibo, China

**Keywords:** alternative splicing, gene regulation, mechanisms, skeletal disorders, therapy

## Abstract

RNA splicing is a fundamental post-transcriptional mechanism that enables the generation of diverse mRNA isoforms from a single gene, thereby expanding proteomic complexity and regulating cell fate decisions. Emerging evidence highlights that dysregulated splicing contributes to the onset and progression of various bone-related diseases, including osteoporosis, osteoarthritis, and skeletal malignancies. In this review, we summarize current knowledge on the core mechanisms of pre-mRNA splicing, with emphasis on alternative splicing events that modulate bone cell differentiation, matrix formation, and tissue homeostasis. We further discuss how aberrant splicing impacts signaling pathways involved in bone metabolism and disease pathogenesis, and we explore the epigenetic and RNA-binding protein networks that fine-tune these processes. Finally, we examine the therapeutic potential of targeting splicing machinery or correcting mis-splicing events using small molecules, antisense oligonucleotides, and RNA-based approaches. This comprehensive overview provides mechanistic insights and highlights splicing regulation as a promising avenue for the diagnosis and treatment of skeletal disorders.

## Introduction

Bone is a dynamic and multifunctional tissue that provides mechanical support, facilitates locomotion, protects vital organs, and regulates mineral homeostasis and hematopoiesis. Its development and continuous remodeling are orchestrated by tightly controlled gene expression programs governed by the coordinated actions of osteoblasts, osteoclasts, and osteocytes (Su et al., 2019). Growing evidence suggests that, in addition to transcriptional control, post-transcriptional mechanisms, most notably RNA splicing, play an essential role in regulating these processes under both physiological and pathological conditions (Zhou et al., 2017).

RNA splicing is a fundamental step in eukaryotic gene expression, whereby introns are removed from precursor mRNA (pre-mRNA) and exons are ligated to generate mature transcripts. This process is catalyzed by the spliceosome, a highly dynamic ribonucleoprotein complex (Black, 2003). Beyond its canonical role, alternative splicing (AS) expands proteomic diversity by producing multiple mRNA isoforms from a single gene. Such splicing-dependent plasticity is particularly vital in complex tissues such as bone, which must integrate a wide range of developmental, metabolic, and mechanical signals (Milona et al., 2003).

In skeletal tissues, AS modulates the expression and function of numerous critical factors, including transcription regulators, signaling molecules, and extracellular matrix components. For instance, the type I collagen genes *COL1A1* and *COL1A2*, which are indispensable for maintaining bone matrix structure and strength, undergo alternative splicing that influences their biophysical and biochemical properties (El-Gazzar et al., 2021; Xia et al., 2008). Similarly, splicing variants of growth factor receptors, ion channels, and osteogenic regulators have been implicated in diverse aspects of bone formation and remodeling (Fan and Tang, 2013).

Disruption of normal splicing patterns is increasingly recognized as a contributor to a variety of bone-related disorders. Mutations affecting canonical splice sites in collagen genes are causatively linked to osteogenesis imperfecta (Stover et al., 1993; Su et al., 2019). Genetic polymorphisms that alter the expression or activity of splicing regulatory proteins have been associated with increased susceptibility to osteoporosis (Langdahl et al., 2002). Furthermore, global splicing aberrations have been documented in osteosarcoma, implicating dysregulated splicing in tumor initiation, progression, and therapy resistance (Dai et al., 2023).

The advent of high-throughput RNA sequencing and single-cell transcriptomics has enabled comprehensive profiling of splicing landscapes in bone cells (Hojo et al., 2016; Hojo and Ohba, 2020). These technologies have uncovered numerous previously unrecognized splicing isoforms, disease-associated splicing events, and essential roles of RNA-binding proteins in maintaining the integrity of the splicing program (Hakelien et al., 2014).

Collectively, these findings position RNA splicing as a central regulatory node in skeletal development, maintenance, and pathology. This review provides a comprehensive overview of RNA splicing mechanisms in bone biology, elucidates their contributions to bone-related diseases, and highlights emerging therapeutic strategies targeting the splicing machinery to restore skeletal health.

## RNA splicing mechanisms

### Pre-mRNA splicing

**FIGURE 1:**
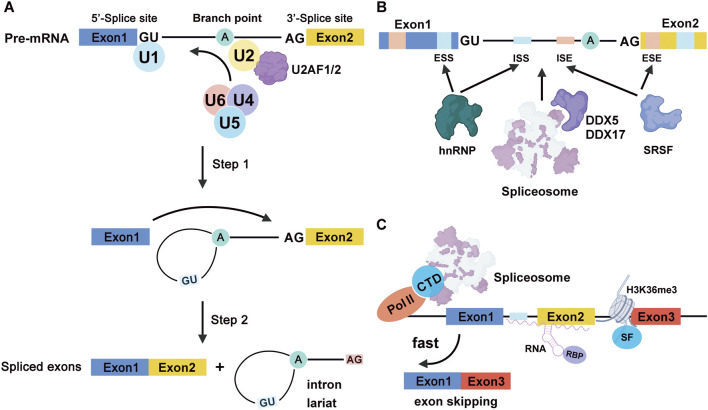
Mechanisms and Regulation of Pre-mRNA Splicing. **(A)** The spliceosome (U1, U2, U4/U6·U5) catalyzes intron removal through two transesterification steps after 5′ splice site and branch point recognition. The first reaction forms the intron lariat, and the second ligates the exons and releases the lariat. **(B)** AS Regulation is mediated by cis-regulatory elements and trans-acting factors, such as SR proteins and hnRNPs, influenced by RNA structure and protein modifications. **(C)** Co-transcriptional splicing is influenced by RNA polymerase II kinetics and chromatin features. The Pol II C-terminal domain recruits spliceosomal components, while slower transcription elongation favors inclusion of alternatively spliced exons. Histone modifications, such as H3K36me3, promote exon inclusion by facilitating binding of splicing regulatory proteins.

Pre-mRNA splicing is a fundamental and highly conserved process that removes introns and joins exons in precursor mRNAs to form mature transcripts. This reaction is catalyzed by the spliceosome, large ribonucleoprotein complex composed of five small nuclear ribonucleoproteins (snRNPs)—U1, U2, U4, U5, and U6—along with numerous associated protein cofactors (Black, 2003; Kinlaw et al., 1982; Sperling et al., 1986; Vagner et al., 2000). The splicing process begins with U1 snRNP recognizing the 5′ splice site and U2 snRNP binding the branch point sequence, facilitated by U2 auxiliary factors U2AF1 and U2AF2 (Glasser et al., 2022; Kralovicova and Vorechovsky, 2017). During spliceosome activation, the subsequent recruitment of the U4/U6·U5 tri-snRNP complex facilitates the structural rearrangements required for catalysis, enabling two sequential transesterification reactions. In the first catalytic step, the 2′-OH of the branch point adenosine attacks the 5′ splice site, generating a cleaved 5′ exon and forming the intron lariat structure through a 2′–5′ phosphodiester bond (Figure 1A). The spliceosome primarily recognizes canonical GT-AG splice site motifs. Mutations within these sites can activate cryptic splice sites or cause exon skipping and intron retention, frequently resulting in aberrant or truncated protein products (Corvelo et al., 2010; Douglas and Wood, 2011; Pros et al., 2008).

### Alternative splicing and its regulation

Alternative splicing (AS) enables a single gene to produce multiple mRNA and protein isoforms by varying the combination of exons incorporated into the final transcript, thereby substantially increasing proteomic complexity without altering the genomic sequence (Tao et al., 2024). Common AS patterns include exon skipping, use of alternative 5′ or 3′ splice sites, intron retention, and mutually exclusive exon usage. It is estimated that approximately 75% of alternatively spliced exons encode regions that contribute to protein functional domains, often affecting surface-exposed residues important for molecular interactions (Blencowe, 2006; Romero et al., 2006).

Splice site selection is governed by the strength of core splicing signals and the presence of cis-regulatory elements, exonic and intronic splicing enhancers and silencers, which serve as binding platforms for trans-acting splicing factors (Figure 1B). Serine/arginine-rich splicing factors (SRSFs) typically enhance exon inclusion by binding to splicing enhancers, while heterogeneous nuclear ribonucleoproteins (hnRNPs) often repress splicing through silencers, though their functional roles are context-dependent and can be bidirectional (Busch and Hertel, 2012). This flexibility contributes to cell type, and signal-specific splicing programs. Additionally, DEAD-box RNA helicases such as DDX5 and DDX17 modulate RNA secondary structures and assist in spliceosome assembly (Dardenne et al., 2014; Lee et al., 2018). Post-translational modifications, particularly phosphorylation of the RS domains in SR proteins, further influence their binding affinity and protein-protein interactions, thereby fine-tuning splice site selection (Long et al., 2019).

### Splicing and epigenetic crosstalk

Splicing is intimately linked with transcription and chromatin dynamics. The C-terminal domain of RNA polymerase II (Pol II) serves as a scaffold for the recruitment of spliceosomal components, facilitating co-transcriptional splicing (Giono and Kornblihtt, 2020). Transcription elongation rates can influence splice site recognition, with slower elongation favoring inclusion of alternatively spliced exons. Chromatin features, such as nucleosome positioning and histone modifications, also modulate splicing outcomes. For instance, H3K36me3 is enriched at exons and has been shown to promote exon inclusion by recruiting splicing regulatory proteins (Figure 1C) (de Almeida et al., 2011).

RNA secondary structures can further regulate splicing by masking or exposing splicing regulatory elements, as demonstrated in genes such as *MAPT* (tau) and *FN1* (fibronectin) (Muro et al., 1999; Ong et al., 2019). The *FN1* pre-mRNA adopts a specific stem-loop around the EDA exon, positioning the exon splicing enhancer within an exposed loop that facilitates proper splicing. Moreover, small nucleolar RNAs, such as HBII-52, have been implicated in modulating alternative splicing and are associated with genetic disorders like Prader-Willi syndrome (Gallagher et al., 2002). Splicing regulation is also influenced by extracellular signals through modulation of RNA-binding protein activity via phosphorylation and other post-translational modifications, adding yet another layer of dynamic control (Blaustein et al., 2007).

## RNA splicing in bone development and homeostasis

Bone development is a tightly regulated biological process involving the differentiation of mesenchymal stem cells (MSCs) into osteoblasts, followed by matrix synthesis, mineralization, and lifelong remodeling in response to systemic hormones and mechanical stimuli. Recent studies underscore the importance of RNA splicing, particularly AS, as a critical regulatory mechanism that fine-tunes gene expression by generating functionally distinct protein isoforms. Importantly, the functionality of bone cells is not solely determined by gene transcription, but also by the specific splicing patterns of pre-mRNA transcripts.

### Splicing in mesenchymal stem cell differentiation

Genome-wide RNA sequencing (RNA-seq) analyses have revealed dynamic shifts in AS profiles during embryonic stem cell differentiation and their reversion upon cellular reprogramming, suggesting that AS contributes to the establishment and maintenance of cell type–specific gene expression programs, though it may not serve as a primary driver of lineage commitment (Laaref et al., 2020). In MSCs, differential AS patterns are observed between young and aged donors, indicating that splicing regulation plays a role in age-associated alterations in differentiation potential (Peffers et al., 2016).

**FIGURE 2:**
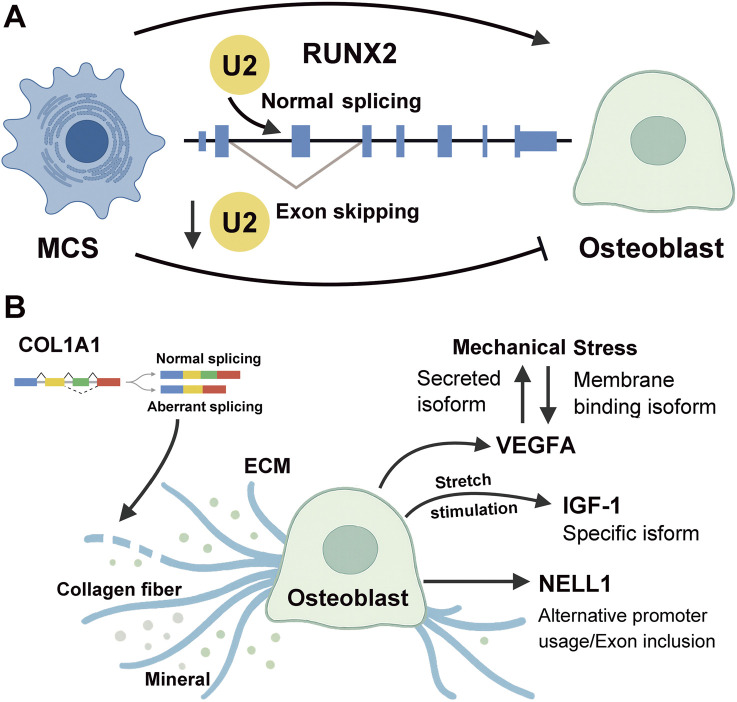
Alternative Splicing in MSC Differentiation and Osteoblast Function. **(A)** AS regulates MSC differentiation by modulating key transcription factors such as RUNX2. Knockdown of U2 snRNP components disrupts RUNX2 splicing, impairing osteogenic commitment. **(B)** In osteoblasts, AS fine-tunes ECM composition and responses to mechanical cues. Splicing of genes such as *COL1A1*, *VEGFA*, *IGF-1*, and *NELL1* influences mineralization and mechanical adaptation, highlighting the functional relevance of AS in bone homeostasis. Mechanical stress reshapes AS profiles; for example, high stress promotes membrane-bound *VEGFA* isoforms, while low stress favors soluble forms. *NELL1* likewise produces two isoforms via alternative promoter usage and exon inclusion.

The transcription factor RUNX2 is a master regulator of osteogenic differentiation, being highly expressed during early lineage commitment and subsequently downregulated in mature osteocytes (Makita et al., 2008). U2 snRNP components such as U2AF1, SF3A1, and SF3A3 are essential for proper splicing of RUNX2 transcripts. Knockdown of these factors induces exon skipping and disrupts RUNX2 function, impairing osteogenic commitment (Venables et al., 2013) (Figure 2A). In parallel, extracellular signaling pathways, including Wnt, BMP, and Notch, modulate AS by altering the expression and activity of splicing factors and RNA-binding proteins.

### Alternative splicing modulates osteoblast function and extracellular matrix dynamics

Osteoblasts are responsible for the production and mineralization of the extracellular matrix (ECM), which is primarily composed of type I collagen, non-collagenous proteins, and hydroxyapatite. AS plays a pivotal role in regulating the structure, composition, and mechanical properties of the ECM. For instance, *COL1A1*, the gene encoding the α1 chain of type I collagen, undergoes AS, and aberrant splicing events are associated with osteogenesis imperfecta (Han et al., 2020).

AS also regulates the activity of osteogenic growth factors such as VEGFA, IGF-1, and NELL1. Mechanical stimulation alters the splicing pattern of *VEGFA*, favoring membrane-bound isoforms under high-stress conditions and soluble isoforms under low stress. This shift affects both mineralization and angiogenesis (Faure et al., 2008). Similarly, mechanical loading induces global AS changes in osteocytes, modulating ECM composition and bone strength. A specific isoform of *IGF-1* is preferentially produced by osteoblasts in response to mechanical stretch, highlighting the mechanosensitive nature of splicing regulation (Xian et al., 2006). *NELL1*, a potent osteoinductive factor, exists as two isoforms generated via alternative promoter usage and exon inclusion. The shorter isoform more efficiently promotes MSC proliferation, although the molecular mechanisms governing its splicing remain poorly understood (Zhang et al., 2010) (Figure 2B).

Together, these findings illustrate that RNA splicing is not merely a passive post-transcriptional process but an active regulatory mechanism essential for maintaining skeletal integrity. By enabling precise temporal and spatial control of gene expression, AS ensures that bone-forming and bone-resorbing activities are balanced in response to developmental and environmental cues.

## Dysregulation of RNA splicing in bone-related diseases

Aberrant RNA splicing has emerged as a pivotal contributor to the pathogenesis of diverse bone-related disorders. Disruptions in splicing can arise from splice site mutations, dysregulated expression or activity of splicing factors, or alterations in the epigenetic landscape governing spliceosome function. These disruptions may result in gain- or loss-of-function isoforms, unstable or nonfunctional transcripts, and altered protein stoichiometry. This section highlights the mechanistic roles of splicing dysregulation in several major bone diseases, including osteogenesis imperfecta, osteoporosis, and osteosarcoma.

### Osteogenesis imperfecta

Osteogenesis Imperfecta (OI) is a heritable connective tissue disorder primarily caused by pathogenic variants in *COL1A1* and *COL1A2*, encoding the pro-α chains of type I collagen. Precise splicing of these genes is critical for the assembly of functional collagen triple helices. Splice site mutations can lead to exon skipping within the triple-helical domain, thereby disrupting chain alignment and compromising helix propagation—mechanisms associated with severe OI phenotypes (Chu and Prockop, 2002). In other cases, splicing alterations activate cryptic splice sites or cause intron retention, leading to premature termination codons and nonsense-mediated mRNA decay, often resulting in milder clinical presentations (Maquat, 2004). These molecular consequences underscore the importance of splicing regulation in both collagen biosynthesis and OI phenotype heterogeneity. Beyond structural defects, altered mRNA splicing impacts transcript stability and collagen stoichiometry, positioning RNA splicing as a central determinant of skeletal integrity.

### Osteoporosis

Osteoporosis is a multifactorial metabolic bone disease characterized by reduced bone mineral density (BMD), microarchitectural deterioration, and increased fracture risk. While aging, hormonal imbalance, and environmental factors are key contributors, genetic and epigenetic mechanisms, including alternative splicing, play substantial roles in disease susceptibility and progression.

In monogenic forms, such as LRP5-related osteoporosis, splice site mutations disrupt exon recognition, resulting in exon skipping, in-frame deletions, or cryptic splice site activation. These mutations yield truncated or nonfunctional LRP5 proteins lacking transmembrane or intracellular domains, impairing Wnt signaling and osteoblast function (Ai et al., 2005; Laine et al., 2011). Notably, some LRP5 splicing mutations are also associated with neurological symptoms, suggesting pleiotropic effects of splicing errors beyond the skeleton.

At a polygenic level, genome-wide association studies (GWAS) and transcriptomic analyses have uncovered widespread splicing alterations correlated with skeletal fragility. A recent meta-analysis identified 32 genes exhibiting splicing events significantly associated with BMD, and an additional 10 genes harboring splicing variants linked to fracture risk (Liu et al., 2020). Among these, *DBF4B* is regulated by the splicing factor SRSF1, which has been identified as a central node in both protein–protein interaction and co-expression networks in postmenopausal osteoporosis (Chen et al., 2017; Qian et al., 2019). These findings underscore RNA splicing as a critical post-transcriptional mechanism shaping the genetic architecture of osteoporosis.

### Osteosarcoma

Osteosarcoma is the most prevalent primary bone malignancy, typically arising during adolescence and characterized by aggressive growth, metastatic potential, and resistance to conventional therapies. Increasing evidence implicates alternative splicing as a driver of osteosarcoma pathogenesis.

High-throughput transcriptome profiling has identified 63 dominant AS events in osteosarcoma tissues, many of which correlate with specific immune cell populations, including resting memory CD4^+^ T cells, dendritic cells, and mast cells. This suggests a role for aberrant splicing in remodeling the tumor immune microenvironment. A regulatory network termed the RBP–AS–immune network has been proposed, linking RNA-binding proteins (RBPs) to immune modulation and cancer progression. Key RBPs—such as NOP58, FAM120C, DYNC1H1, TRAP1, and LMNA—emerge as promising targets for therapeutic immunomodulation (Dai et al., 2023).

Among splicing regulators, SRSF3 displays oncogenic properties, and its overexpression has been shown to drive tumor formation in nude mice (Jia et al., 2010). Transcriptome-wide studies in U2OS cells demonstrate that SRSF3 modulates splicing and expression of over 200 genes involved in cell cycle control and proliferation. Its knockdown also alters microRNA expression, suggesting broader roles in gene regulatory networks (Ajiro et al., 2016).

AS also governs the isoform expression of vascular endothelial growth factor (VEGF), a key modulator of tumor angiogenesis. The proangiogenic isoform VEGF_165_ is upregulated in osteosarcoma, while the antiangiogenic isoform VEGF_165_b is downregulated. The splicing factor YBX1, overexpressed in osteosarcoma, promotes VEGF_165_ expression and represses VEGF_165_b, thereby enhancing tumor cell proliferation, migration, and invasion. High YBX1 expression correlates with poor prognosis (Bates et al., 2002; Quan et al., 2023). These findings highlight splicing regulators as potential therapeutic targets for osteosarcoma.

### Other bone-related disorders associated with splicing defects

Several rare skeletal disorders also implicate RNA splicing in their pathogenesis.

Odontochondrodysplasia (ODCD): ODCD is a rare skeletal dysplasia characterized by short stature, joint laxity, craniofacial abnormalities, and dental defects, including dentinogenesis imperfecta (Wehrle et al., 2019). A homozygous in-frame splicing mutation in intron 9 of *TRIP11* leads to the expression of an alternative transcript, likely contributing to the mild skeletal phenotype and dentinogenesis imperfecta characteristic of ODCD (Medina et al., 2020).

Marfan Syndrome (MFS): MFS is a systemic connective-tissue disorder presenting with aortic root dilation, ectopia lentis, long-bone overgrowth, and skeletal abnormalities such as scoliosis and pectus deformities. Pathogenic splicing mutations in *FBN1* have been identified as causative of MFS. A recently reported donor site mutation (c.8051+1G>C) in exon 64 disrupts canonical splicing, leading to disease development (Comeglio et al., 2007; Karttunen et al., 1998; Wang et al., 2022).

X-linked Spondyloepiphyseal Dysplasia Tarda (SEDT): Caused by splice-disrupting mutations in *SEDL*, this disorder is characterized by short stature and joint degeneration. Splicing errors in *SEDL* result in abnormal protein isoforms, impairing intracellular trafficking (Shaw et al., 2003; Xiong et al., 2009).

Paget’s Disease of Bone (PDB): PDB is a chronic focal bone disorder characterized by markedly increased and disorganized bone turnover, leading to bone pain, deformity, and a heightened risk of fractures, particularly in the pelvis, spine, skull, and long bones (Hasebe and Hamasaki, 2025). RNA-seq studies identified six AS events associated with PDB in genes such as *LGALS8*, *RHOT1*, *CASC4*, *USP4*, *TBC1D25*, and *PIDD*, suggesting potential roles for splicing defects in altered bone remodeling (Klinck et al., 2014).

## Therapeutic potential of targeting RNA splicing in bone diseases

Aberrant RNA splicing is increasingly recognized as a critical driver of numerous bone-related diseases, including skeletal dysplasias and bone tumors. Therapeutic strategies that target splicing aim to correct disease-causing splicing defects, modulate isoform expression, or exploit dependencies in the splicing machinery unique to pathological cells. Mutations affecting the 5′ splice site, 3′ splice site, or branch point sequence can severely disrupt canonical splicing, necessitating precise, mutation-specific interventions. Several emerging therapeutic modalities have shown promise in preclinical or unrelated clinical contexts and may be applicable to bone disorders with analogous splicing defects.

### Antisense oligonucleotides

**FIGURE 3:**
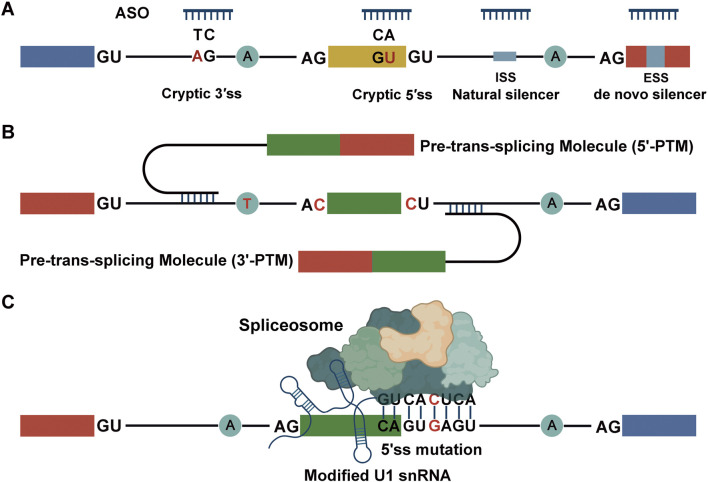
Therapeutic Potential of Targeting Aberrant Splicing Caused by Mutations. **(A)** Antisense oligonucleotides (ASOs, shown in blue) are designed to bind specifically to pre-mRNA sequences to modulate splicing. They can mask mutation sites (red) that would otherwise disrupt normal splicing, or target splicing silencers or enhancers, thereby restoring correct exon inclusion or exclusion. By sterically blocking access of the spliceosome or splicing regulatory proteins to these sites, ASOs redirect the splicing machinery to produce properly spliced mRNA, correcting the functional consequences of pathogenic mutations. **(B)** Trans-splicing rescue of aberrant transcripts. A pre–trans-splicing molecule (PTM) replaces the mutated 3′ or 5′ portion of the pre-mRNA with a wild-type sequence, thereby restoring functional splicing. Core splicing sequence mutations are indicated in red. **(C)** Engineered U1 snRNA molecules carry compensatory base changes that restore proper base-pairing with mutated 5′ splice sites (red) in pre-mRNA. By re-establishing this interaction, the modified U1 snRNA facilitates recognition of the affected exon by the spliceosome, promoting correct exon inclusion and normal splicing.

Antisense oligonucleotides (ASOs) are short, synthetic, single-stranded nucleic acids designed to modulate pre-mRNA splicing by binding to specific RNA sequences. They can restore normal splicing patterns by masking aberrant splice sites, blocking splicing silencers or enhancers, or promoting exon skipping or inclusion (Figure 3A). In cases where pathogenic mutations activate cryptic splice sites or disrupt exon definition, ASOs can redirect splicing to restore the correct reading frame or produce partially functional proteins.

While ASO-based therapy has not yet been applied to bone diseases, its clinical success in correcting a 3′ splice site mutation in the *DMD* gene for Duchenne muscular dystrophy highlights its therapeutic potential in disorders with similar molecular mechanism (Yokota et al., 2011). ASOs can also sterically block pseudo splice sites induced by mutation, thereby promoting usage of authentic splice sites (Uchikawa et al., 2007). Pharmacological agents such as kinetin have also demonstrated splicing modulation capabilities. For instance, kinetin partially restores correct splicing at a mutated 5′ splice site in the *IKBKAP* gene in familial dysautonomia (Axelrod et al., 2011). These findings support the plausibility of pharmacological or ASO-mediated splicing correction in bone disorders with analogous splice site mutations.

### Trans-splicing

Trans-splicing, or spliceosome-mediated RNA trans-repair, offers a powerful strategy for correcting endogenous mRNA at the RNA level. This approach utilizes pre–trans-splicing molecules (PTMs) to replace mutated regions of a pre-mRNA with a wild-type sequence via splicing-mediated ligation (Figure 3B). PTMs contain an RNA binding domain specific to the target transcript, a functional splice site, and the corrective coding sequence.

This technique is particularly suited for correcting mutations at canonical splice sites and has shown efficacy in preclinical models of β-thalassemia by repairing *HBB* gene mutations (Berger et al., 2016; Kierlin-Duncan and Sullenger, 2007). Although trans-splicing has not yet been explored in bone-related conditions, its successful application to similarly structured genes suggests its potential translational relevance for skeletal diseases.

### Modified snRNAs

Engineered small nuclear RNAs (snRNAs), particularly modified U1 snRNAs, represent another promising modality for rescuing splicing defects. U1 snRNAs recognize the 5′ splice site during early spliceosome assembly. Introducing compensatory base changes in U1 snRNA can restore complementarity to mutated 5′ splice sites, thereby facilitating accurate exon recognition and inclusion (Bohnsack and Sloan, 2018) (Figure 3C). This strategy is broadly applicable to splice site mutations, particularly those that disrupt base pairing without compromising the core splicing machinery. However, mutations at the highly conserved first and second intronic nucleotides are challenging to rescue due to their essential catalytic roles (Fernandez et al., 2012; Glaus et al., 2011). Nevertheless, advancements in snRNA design may overcome these obstacles and expand their utility in bone disease treatment.

Collectively, these splicing-targeted approaches offer promising therapeutic avenues for bone disorders driven by splice site mutations. Although most of these strategies remain in preclinical stages for skeletal disorders, their proven efficacy in other genetic diseases underscores their translational potential. Further research and disease-specific validation will be essential to bring these RNA-based therapeutics into clinical application for bone pathologies.

## Discussion

RNA splicing is rapidly emerging as a pivotal regulator of skeletal biology, modulating processes that span stem cell differentiation, mechanotransduction, bone remodeling, and disease progression, including tumorigenesis (Chabot and Shkreta, 2016; Lee and Rio, 2015). Despite increasing recognition of its fundamental roles, research focused on splicing regulation in bone remains relatively nascent, with several critical gaps yet to be addressed.

First, comprehensive and high-resolution splicing maps specific to bone tissues are lacking. Unlike well-characterized tissues such as brain or blood, bone presents unique challenges due to its marked cellular heterogeneity, encompassing osteoblasts, osteocytes, osteoclasts, and marrow stromal cells (Arora and Robey, 2022). The application of cutting-edge technologies, such as single-cell and spatial transcriptomics coupled with long-read sequencing platforms like Oxford Nanopore (Weirather et al., 2017) or PacBio (Rhoads and Au, 2015), holds great promise to elucidate novel isoforms and cell type–specific alternative splicing programs, particularly those associated with skeletal diseases or regenerative processes.

Second, the contribution of splicing regulation within the bone marrow niche and its impact on hematopoietic cell development remain underexplored. Given the critical role of this microenvironment in immune regulation, osteoclastogenesis, and hematological malignancies such as multiple myeloma, deeper insights into splicing dynamics here could reveal novel therapeutic targets (An et al., 2016; Mansour et al., 2017). Similarly, mechanotransduction—how mechanical forces influence alternative splicing in osteocytes and osteoblasts—represents a promising yet insufficiently investigated area vital to understanding bone adaptation.

Age and sex significantly affect bone physiology, yet systematic profiling of splicing changes across age groups and between sexes is absent. Deciphering how hormonal changes and aging impact RNA splicing may facilitate the development of tailored therapies for disorders such as postmenopausal osteoporosis and age-related bone loss. Moreover, splicing-derived biomarkers, including circulating cell-free and exosomal RNAs, are emerging as valuable tools for non-invasive diagnosis and disease monitoring in bone malignancies like osteosarcoma (Fanale et al., 2018; Liu et al., 2020).

From a therapeutic standpoint, RNA splicing modulation faces challenges related to delivery efficiency, target specificity, and safety (Kaczmarek et al., 2017; Sune-Pou et al., 2020). Innovations such as tissue-specific promoters, RNA-guided delivery systems, and nanoparticle carriers are being developed to enhance targeting precision, while computational modeling and machine learning approaches are improving the prediction of splicing outcomes and minimizing off-target effects. The integration of splicing biology with systems medicine frameworks will be critical to successfully translate these advances into clinical practice.

In summary, RNA splicing constitutes a sophisticated layer of gene regulation that amplifies the functional diversity of bone cells. Recent discoveries have elucidated its integral roles in skeletal development, extracellular matrix synthesis, and pathogenesis of bone diseases, including genetic disorders, osteoporosis, and cancer (El-Gazzar et al., 2021; Langdahl et al., 2002; Quan et al., 2023; Zhang et al., 2010). Technological advancements in sequencing and molecular biology have begun to unveil the bone-specific splicing landscape, while emerging therapeutic tools such as antisense oligonucleotides (O'Callaghan et al., 2020) and CRISPR-based editing technologies (Modell et al., 2022) are advancing the prospect of precise splicing modulation.

Nevertheless, the inherent complexity of the splicing machinery, combined with the structural and cellular diversity of bone, presents both formidable challenges and unique opportunities. Ongoing interdisciplinary collaboration among molecular biologists, clinicians, bioengineers, and computational scientists will be essential to fully harness splicing mechanisms for improved diagnostics, regenerative therapies, and personalized treatments of skeletal diseases. As our understanding deepens, RNA splicing is poised to transform the field of bone biology and open novel avenues for maintaining skeletal health.
